# Three novel mutations of the G6PC gene identified in Chinese patients with glycogen storage disease type Ia

**DOI:** 10.1007/s00431-014-2354-y

**Published:** 2014-07-01

**Authors:** Bi-Xia Zheng, Qian Lin, Mei Li, Yu Jin

**Affiliations:** 1 Nanjing Children’s Hospital Affiliated to Nanjing Medical University, Nanjing, China; 2 Nanjing Children’s Hospital, Medical School of Nanjing University, Nanjing, China

**Keywords:** GSDIa, Glucose-6-phosphatase, Germline mutation

## Abstract

Glycogen storage disease type Ia (GSDIa) is an autosomal recessively inherited disease characterized by poor tolerance to fasting, growth retardation, and hepatomegaly resulting from accumulation of glycogen and fat in the liver. Germline mutations of glucose-6-phosphatase (G6PC) gene have been identified as a cause of GSDIa. In this study, we performed mutation analysis in five Chinese GSDIa patients belonging to five unrelated families by direct DNA sequencing. All patients were clinically classified as GSDIa. Mutation analysis of the G6PC gene revealed that all patients carried biallelic G6PC mutations (p.Ile341Asn, p.Ala274Val, p.Phe80Ile, p.Gly118Asp, p.Arg83His, c.262delG, and c.648G>T). Of the seven different mutations identified, three were found to be novel. All of the novel mutations were missense (p.Ala274Val, p.Phe80Ile, and p.Gly118Asp). The c.262delG mutation which leads to a frame-shift and truncated forms of glucose-6-phosphatase was present in three unrelated patients (one homozygote and two heterozygotes). *Conclusion*: By direct DNA sequencing, three novel G6PC variations were identified which expanded the G6PC mutation spectrum, and provided conclusive genetic evidences for the definitive diagnosis of the Chinese patients.

## Introduction

Glycogen storage diseases (GSD) are a group of inherited metabolic disorders of glycogen metabolism. There are over 12 different types, and they are classified based on the deficient enzymes and affected tissues [10]. The overall incidence of GSD is estimated at 1:20,000–40,000 cases per live birth [2]. The most common type with hepatic involvement is GSD I, including types Ia (GSDIa; MIM232200) and Ib (GSDIb; MIM232220). GSDIa is the more frequent type, representing about 80 % of GSD I patients [5]. GSDIa is an autosomal recessively inherited disease characterized by poor tolerance to fasting, growth retardation, and hepatomegaly resulting from accumulation of glycogen and fat in the liver [3].

GSDIa is caused by the deficiency of the glucose-6 phosphatase activity [9]. Human *G6PC* gene has been localized to chromosome 17 at 17q21, spans 12.5 kb, includes 5 exons, and codes for a highly hydrophobic protein of 357 amino acids containing 9 transmembrane helixes [11]. To date, more than 105 different *G6PC* germline mutations have been characterized worldwide demonstrating allelic heterogeneity.

With DNA-based genetic techniques, GSDIa can be diagnosed with DNA extracted from peripheral blood. *G6PC* mutational analysis can spare the patients from invasive liver biopsy and facilitate family screening and is essential for prenatal diagnosis of GSDIa. In this study, we report the clinical and genetic findings of five probands and their parents.

## Patient and methods

### Patient and sample collection

Five Chinese patients from five unrelated families with a clinical diagnosis of GSDIa were investigated. All of them come from Jiangsu province. In all patients, diagnosis was based on clinical symptoms and laboratory findings. After informed consent had been obtained, genomic DNA was extracted from peripheral blood samples for molecular genetic analysis of the *G6PC* gene. The study protocol was approved by the ethics committee of Nanjing Children’s Hospital Affiliated to Nanjing Medical University.

### Genomic DNA isolation and polymerase chain reaction

Genomic DNA of peripheral blood leucocytes was extracted routinely by isolation kit (Tiangen, China) according to the manufacturer’s instructions. All five coding exons and flanking introns of the *G6PC* gene were amplified by the use of primers listed in Table 1. PCRs of G6PC exons were performed in a 50 μl reaction which contained 1× PCR buffer, 0.2 mmol/L dNTPs, 0.4 μL mol/L of each primer, 50 ng genomic DNA, and 1 U Tag DNA polymerase (Takara). The PCR were performed under the following conditions: denaturation at 95 °C for 4 min, followed by 40 thermal cycles, each composed of 95 °C for 30 s, at 58 °C for 30 s, and at 72 °C for 45 s.

**Table 1:** Primers for exon-specific sequencing of G6PC gene

Exon	Forward primer	Reverse primer	Product length (bp)
Exon1	TCAACCTACTGGTGATGCAC	GATCCAAAGTCAGAGAGAGG	586
Exon2	ATCCTTCTCAGGCTACACTC	GCCAATTGAAGAGCATGAC	328
Exon3	ATTCCCAGAGCCTTGCACAA	CCCTATCCAAAGTGCCACAA	504
Exon4	CCAACAGGCATCTTTGGACT	GATTACACACAGGATGTGGC	378
Exon5	CTAGCAAAGGTCCCAAATCC	TTGCTCCAAATACCAGTGCC	662

### DNA sequencing

The PCR products were gel- and column-purified and directly sequenced. The purified PCR fragments were then sequenced using BigDye Terminator (Applied Biosystems, Foster City, CA, USA) on an ABI Prism 3100 genetic analyzer (Applied Biosystems). In addition, samples from 50 unrelated healthy controls were sequenced for novel missense mutations to exclude the mutations as non-disease associated variations in the Chinese population.

### Pathogenicity analysis of the novel mutations

Proof of pathogenicity in this study was defined by at least one of the following criteria: (1) a mutation presenting at the frequency of <1 % in at least 50 unrelated healthy controls, (2) a mutation with co-segregation in a family, (3) alteration of an evolutionary conserved amino acid residue, and (4) nonsense and deletion variation in the coding sequence of G6PC gene. Moreover, the function effect of the novel missense mutations identified in this study was predicted with the software PolyPhen-2 (http://genetics.bwh.harvard.edu/pph2/), and a mutation is classified as “probably damaging” if its probabilistic score is above 0.85 while as “possibly damaging” with the score above 0.15.

## Results

### Clinical and genetic findings

Clinical findings of the patients are presented in Table 2. All patients had hepatomegaly, fasting hypoglycemia, fasting lactic acidosis, and hyperlipidemia. None of the patients had severe neutropenia. None of the patients had severe recurrent infections. Four of five unrelated patients were compound heterozygotes for *G6PC* mutations. We observed a homozygous c.262delG mutation in one patient from a nonconsanguineous family.

**Table 2:** Clinical and genetic findings of five Chinese GSDIa patients

Patient no.	Sex	Onset year-old	Fasting hypoglycemia	Fasting lactic acidosis	Growth retardation	Hepatomegaly	Hyperlipidemia	Hyperuricaemia	G6PC genotypes		
									Patient	Father	Mother
1	M	2 years	+	+	+	+	+	−	p.Ala274Val/p.Ile341Asn	p.Ala274Val	p.Ile341Asn
2	F	3 years	+	+	−	+	+	−	p.Arg83His/p.Phe80Ile	p.Arg83His	p.Phe80Ile
3	M	4 months	+	+	−	+	+	+	c.262delG/c.262delG	–	c.262delG
4	M	4 months	+	+	−	+	+	−	c.262delG/p.Gly118Asp	p.Gly118Asp	c.262delG
5	M	6 months	+	+	−	+	+	−	c.262delG/c.648 G>T	c.262delG	c.648 G>T

+ symptom present; − symptom absent

### Novel missense mutations

**Fig. 1:**
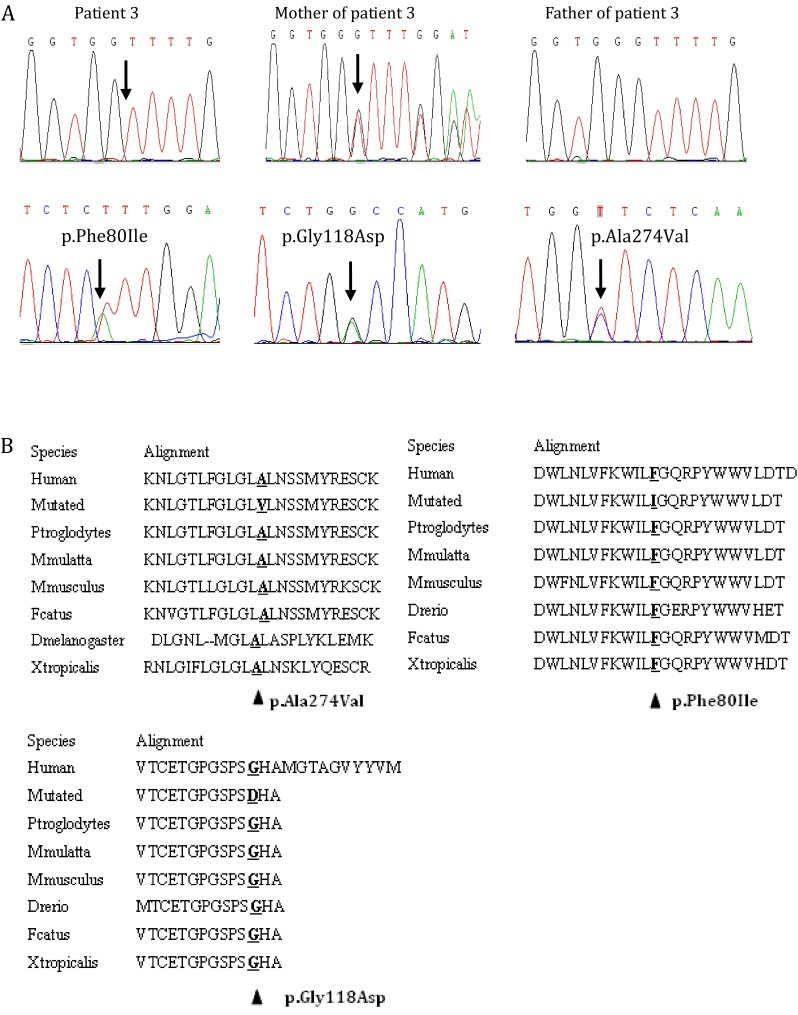
**a** Results of *G6PC* sequence analysis. *Arrow* indicates mutation sites. The sequence analysis of the patient 3 showed a 1 bp homozygous deletion (c.262delG) mutation in exon 2. His mother was heterozygous for c.262delG. This allelic variant was not observed in his father. **b** Comparative alignment of the amino acid sequence of G6PC. Amino acid conservation is indicated by *gray highlights*

Three novel missense mutations (p.Ala274Val, p.Phe80Ile, and p.Gly118Asp) were identified by direct DNA sequencing analysis of the five exons and their flanking sequences in *G6PC* gene (Fig. 1a). The frequency of the missense mutations was investigated in 50 healthy controls by sequencing approach, and all of them proved to be 0 % (Table 3). In addition, comparative alignment of the amino acid sequence of *G6PC* in different species with human *G6PC* further documented the conservative properties of the amino acids involved by the three missense mutations (Fig. 1b). And, on PolyPhen-2 analysis of their function effect, p.Ala274Val and p.Gly118Asp were “possibly damaging” with both a score under 1.00 while the remaining one p.Phe80Ile “probably damaging,” with a score of 1.00. These evidences documented that the novel missense mutations identified in this study were all disease-causing.

**Table 3:** Mutations in the *G6PC* gene identified in five Chinese GSDIa patients

Type	Exon	Nucleotide change	Amino acid change	Domain	Frequency (%)
*Missense*	*Exon 2*	*c.238T*>*A*	*p.Phe80Ile*	*Helix*-*2*	*0* %
Missense	Exon 2	c.248G>A	p.Arg83His	Luminal loop-1	–
Frameshift	Exon 2	c.262delG	p.Val88FfsX14	Luminal loop-1	–
*Missense*	*Exon 3*	*c.353G*>*A*	*p.Gly118Asp*	*Luminal loop*-*1*	*0* %
*Missense*	*Exon 5*	*c.821C*>*T*	*p.Ala274Val*	*Helix*-*7*	*0* %
Missense	Exon 5	c.1022T>A	p.Ile341Asn	Helix-9	–
Splicing mutation	Exon 5	c.648G>T	no AA change	Helix-6	–

Novel mutations are shown in italics

## Discussion

Since *G6PC* gene was cloned as the causative gene for GSDIa, genetic analysis of this gene had been well-recognized as a reliable tool for the definitive diagnosis of GSDIa patients. By direct DNA sequencing, three novel *G6PC* variations were identified in this paper and all of them have not been reported in the Human Gene Mutation Database (HGMD; http://www.hgmd.cf.ac.uk). Laboratory and bioinformatics analysis demonstrated that these mutants were GSDIa-associated pathogenic mutations. These novel mutations expanded the *G6PC* mutation spectrum, and provided conclusive genetic evidences for the definitive diagnosis of the Chinese patients.

The missense mutation p.Ala274Val and p.Phe80Ile found in this study have not been reported yet, and these mutants are located in the transmembrane helix structure of G6PC. Glucose-6-phosphatase was anchored in the ER membrane by nine transmembrane helix structures. The amino-terminal was positioned in the endoplasmic reticulum lumen, and the carboxy-terminal was located in the cytoplasm [6, 7]. Shieh and Angaroni suggested that the majority of helical missense mutants supported the synthesis of reduced levels of G6PC protein compared to the wild-type enzyme, indicating these mutations destabilize *G6PC* [1, 8]. Shieh also showed that luminal loop 1 may play a crucial role in catalytic activity of the enzyme because T108I, E110K, and P113L mutations, which totally inactivated G6Pase, are located within this loop. The missense mutation p.Gly118Asp found in this study is also located within luminal loop 1. A 1 bp homozygous deletion (c.262delG) mutation in exon 2 was identified in patient 3 (Fig. 1a). This mutation caused a change of the coding exons with a frame-shift and truncated the G6PC protein with the substitution of 102 abnormal amino acid residues. His mother was heterozygous for c.262delG, indicating that she is a carrier of the mutation. This allelic variant was not observed in his father, and is likely to be maternal uniparental disomy.

So far, more than 550 unrelated patients affected with GSDIa have been reported worldwide and over 105 mutations (Human Gene Mutation Database; http://www.hgmd.cf.ac.uk) have been identified in *G6PC* gene coding region. All of them are small gene alterations, including missense mutations, small base deletions and insertions, and splice site mutations, and the majority are missense mutations (64 %). Mutations reduce or even completely abolish G6PC enzymatic activity. *G6PC* gene mutations has certain racial differences [4]: in the Caucasian population, R83C and Q347X are found in 33 and 18 % of the GSDIa alleles, respectively; the most common mutations in Jews is R83C (98 %); Japan and South Korea has the most common c.648G>T mutation (91 and 75 %); in Chinese patients, c.648G>T is also frequent (54 % of the alleles), and p.R83H is present in 26 % of the mutated alleles. The c.262delG mutation which leads to a frame-shift and truncated forms of glucose-6-phosphatase was present in three unrelated patients (one homozygote, two heterozygotes). This result indicates that c.262delG mutation may be a race-specific mutation type, but needs further study with expanded sample size.

This study demonstrated that the G6PC enzyme activity reduction due to the *G6PC* gene mutation was the molecular genetic basis of clinical manifestations of the GSDIa patients. Early diagnosis and positive treatment is of important significance to the GSDIa patients’ prognosis. With the expanding knowledge of the mutation spectra of different patient populations, molecular analysis based on genomic DNA can be a reliable and accurate way of diagnosing GSDIa without invasive liver biopsies for enzyme tests.

## Abbreviations

GSD: Glycogen storage disease
GSDIa: Glycogen storage disease type Ia
*G6PC*: Gene coding for glucose-6-phosphatase
G6Pase: Glucose-6-phosphatase
HGMD: Human gene mutation database
PCR: Polymerase chain reaction
